# Defining proteomic subtypes to understand the heterogeneity of Parkinson's pathophysiology

**DOI:** 10.1002/alz70855_104852

**Published:** 2025-12-24

**Authors:** Rashmi Maurya, Sacha Gandhi, Hanjun Zhao, Alejo J Nevado‐Holgado, Donald G Grosset, Laura M Winchester

**Affiliations:** ^1^ Department of Psychiatry, University of Oxford, Oxford, United Kingdom; ^2^ University of Glasgow, Glasgow, United Kingdom; ^3^ University of Oxford, Oxford, United Kingdom

## Abstract

**Background:**

Proteomic biomarkers can be used to define disease subtypes to better understand PD pathophysiology and heterogeneity. Identification of biomarkers related to cognitive change and motor decline will help researchers study Parkinson's disease (PD) progression.

**Method:**

We analyzed longitudinal proteomic data from the Tracking Parkinson's Cohort, consisting of 4530 samples from 1918 recent‐onset PD patients across 72 UK sites. Samples were measured on the Somalogic platform (7596 proteins) at up to five timepoints, but for robust analysis, we focused on the first three timepoints for 794 patients. Key clinical measures included MoCA (cognition), MDS‐UPDRS‐III (motor scores) and levodopa equivalent daily dose. Using Weighted Gene Co‐Expression Network Analysis (WGCNA), we generated protein co‐expression clusters and performed pathway enrichment analysis. Cluster preservation across timepoints was also assessed. Finally, patient clusters from the same longitudinal proteomic data (first three visits, 794 patients) were generated using AlignedUMAP and HDBSCAN to explore pathology predictive of phenotypes.

**Result:**

Cross‐sectional clustering revealed consistent protein co‐expression modules across timepoints. The largest module, strongly preserved across all three timepoints, was enriched for cytokine‐cytokine receptor interaction pathways and showed the strongest negative correlation with age (*r* = ‐0.12, ‐0.14, ‐0.17 and *p*‐value = 5.51x10^‐4^, 5.92x10^‐5^, 7.77x10^‐7^ across timepoints). Module preservation analysis indicated high preservation for most modules, with the largest having the highest preservation (Zsummary > 30). Pathway enrichment analysis also uncovered novel metabolite pathways in modules with dynamic protein expression changes. Patient orientated clustering identified three clusters of disease progression with distinct clinical phenotypes for movement and disease duration.

**Conclusion:**

Our analysis identifies potential proteomic subtypes that enhance our understanding of PD heterogeneity. The largest preserved module, enriched for cytokine interactions, along with another module highlighting novel metabolite pathways, may reveal insights into disease progression and clinical variability. Detailed characterisation of these proteomic clusters will provide a more comprehensive view of PD progression offering deeper insights into PD pathophysiology.

